# Dedicated Breast PET-Based Deep Learning Radiomics for Prediction of Pathologic Complete Response to Neoadjuvant Chemotherapy in HER2-Positive Breast Cancer

**DOI:** 10.3390/cancers18101581

**Published:** 2026-05-13

**Authors:** Tianhao Zeng, Yilin He, Teng Zhang, Caiyue Ren, Jun Xu, Jingyi Cheng, Wenlong Ming

**Affiliations:** 1 Jiangsu Key Laboratory of Intelligent Medical Image Computing, School of Artificial Intelligence, Nanjing University of Information Science and Technology, 219 Ning Liu Road, Nanjing 210044, China; thzeng@nuist.edu.cn (T.Z.); zhangteng@nuist.edu.cn (T.Z.); jxu@nuist.edu.cn (J.X.); 2 Department of Nuclear Medicine, Shanghai Proton and Heavy Ion Center, Fudan University Cancer Hospital, Shanghai Key Laboratory of Radiation Oncology, Shanghai Engineering Research Center of Proton and Heavy Ion Radiation Therapy, Shanghai 201315, China; yilinhe_fudan@163.com (Y.H.); caiyue.ren@sphic.org.cn (C.R.)

**Keywords:** dedicated breast PET, HER2-positive breast cancer, neoadjuvant chemotherapy, deep learning radiomics, pathologic complete response prediction

## Abstract

**Objectives:** To exploratorily evaluate the potential of baseline dedicated breast PET (D-PET) for noninvasive prediction of pathological complete response (pCR) to neoadjuvant chemotherapy (NAC) in HER2-positive (HER2+) breast cancer, and to investigate a fusion strategy integrating conventional radiomics and deep learning features. **Methods:** We developed a multi-representation framework with radiomics based on data-driven high-/low-uptake metabolic subregions and deep learning trained on standardized 3D tumor volumes, and intratumoral heterogeneity (ITH) was quantified on the largest slice as an additional comparator. The outputs of these pathways were subsequently integrated through feature-level and decision-level fusion. Model performance was evaluated using the area under the receiver operating characteristic curve (AUC), and interpretability analyses were applied to identify image regions and features contributing to predictions. **Results:** In a HER2-positive breast cancer cohort (*n* = 147) with baseline D-PET, deep learning (3D ResNet, AUC = 0.79) and radiomics (logistic regression, AUC = 0.78) achieved comparable performance on the primary test set, whereas the ITH model showed limited value (AUC = 0.61). Fusion further improved discrimination on test set 1, with an AUC of 0.83 for decision-level fusion and 0.84 for feature-level fusion. On test set 2, decision-level fusion achieved the highest AUC (0.84), and feature-level fusion maintained stable performance (AUC = 0.80). **Conclusions:** In this exploratory study, baseline D-PET showed promising performance for noninvasive prediction of NAC response in HER2+ breast cancer. The fusion of deep learning and radiomics yielded improvements over single-representation models, highlighting the potential role of D-PET models as decision-support tools.

## Simple Summary

Patients with HER2-positive breast cancer often receive neoadjuvant chemotherapy before surgery, but doctors still lack reliable noninvasive tools to predict who will achieve a pathologic complete response. In this study, we explored whether baseline dedicated breast PET imaging could help address this problem. We developed and compared several imaging-based approaches, including radiomics, deep learning, and fusion models that combined both strategies. Our results showed that baseline dedicated breast PET provided useful information for treatment-response prediction, and the combined models achieved more balanced and accurate performance than single-method models alone. These findings suggest that dedicated breast PET, together with artificial intelligence, may support earlier and more individualized treatment planning for patients with HER2-positive breast cancer.

## 1. Introduction

Breast cancer is the most common malignancy among women. Among its subtypes, HER2-positive (HER2+) breast cancer accounts for 15–20% of cases [1]. HER2 overexpression confers tumor aggressiveness, and although anti-HER2 therapies have improved outcomes [2], prognosis is relatively poor—worse than Luminal A and Luminal B subtypes, but better than triple-negative breast cancer. Importantly, approximately 60% of HER2+ patients achieve pathological complete response (pCR), a strong surrogate for favorable long-term prognosis [3,4,5], underscoring the clinical value of identifying likely pCR achievers upfront.

Dedicated breast PET (D-PET) is a breast-focused molecular imaging technique that uses ^18^F-FDG or other tracers. Compared with ultrasound or MRI, it is less influenced by breast density or menopausal status, yields fewer false positives, and detects metabolic abnormalities earlier [6,7]. It also offers higher spatial resolution than whole-body PET-CT, better characterizing FDG uptake patterns, an important predictive marker [8], and shows higher sensitivity for ductal carcinoma in situ and microinvasive lesions (<1 cm) [9]. Together, these properties suggest that D-PET can provide subtype-relevant metabolic cues for early NAC (neoadjuvant chemotherapy) response prediction in HER2+ disease.

Currently, pCR confirmation relies on postoperative histopathology, which is invasive and time-consuming. Conventional response assessment by RECIST [10] is limited by subjectivity, underscoring the need for baseline imaging biomarkers to predict response early and guide therapy. Most NAC prediction studies target unselected or triple-negative populations, with few focused on HER2+. To address this gap, we develop and validate D-PET based imaging models for early prediction of NAC response in HER2+ breast cancer.

Imaging-based NAC response prediction has been investigated using multiple modalities, including ultrasound, CT, MRI, PET/CT, and functional metabolic imaging [11,12,13,14,15]. Quantitative imaging approaches, particularly radiomics and deep learning, have become major strategies for pCR prediction and have been widely applied in breast cancer imaging for diagnosis, response prediction, axillary status assessment, and prognosis [16,17,18,19]. Radiomics converts tumor intensity, shape, and texture patterns into structured quantitative descriptors with relatively good interpretability and sample efficiency, whereas deep learning can automatically learn high-level semantic and nonlinear image representations directly from volumetric data. Nevertheless, the performance of existing NAC prediction models varies by molecular subtype and may be reduced in HER2+ tumors [20,21,22]. Moreover, many prior studies enrolled mixed molecular subtypes, focused on non-HER2-positive populations, or used conventional imaging modalities such as MRI, ultrasound, or whole-body PET/CT. These limitations motivate exploration of D-PET-based imaging representations and modeling strategies that better capture HER2+ specific tumor characteristics.

Recently, deep learning radiomics strategies suggest that combining handcrafted quantitative features with automatically learned image representations may improve prediction compared with either approach alone [23], particularly in small-sample medical imaging settings where model robustness and interpretability are both important. Therefore, in contrast to prior studies that mainly relied on single-representation models, we compared fusion frameworks based on intermediate fusion (feature-level fusion) and late fusion (decision-level fusion) to investigate whether radiomics and deep-learning representations could jointly exploit complementary D-PET information for pCR prediction in HER2-positive breast cancer.

**Figure 1:**
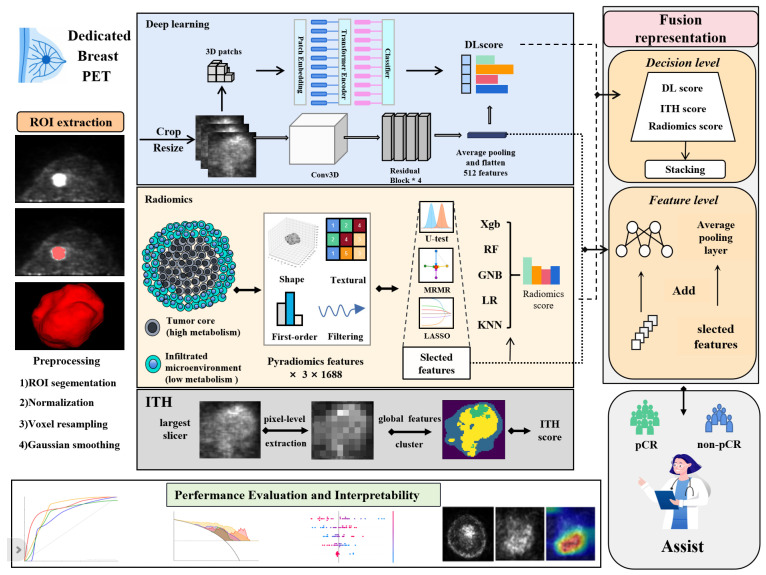
Overall workflow of the study. D-PET images were acquired and tumor regions of interest (ROIs) were delineated. Metabolic subregions were first obtained via clustering; radiomics features were then extracted from the whole ROI and from these clustered subregions, followed by feature selection and classification to generate a radiomics score. In parallel, deep learning prediction was performed using a 3D ResNet architecture on preprocessed images to obtain a deep learning score. Model fusion strategies were employed at the feature level and decision level to integrate the results from both approaches for optimized prediction of NAC response.

In this context, we hypothesized that baseline D-PET encodes discriminative intra- and peri-tumoral metabolic information sufficient for early prediction of pCR in HER2+ breast cancer. Using a subtype-specific D-PET cohort for NAC response prediction in this subtype, we developed three complementary streams: conventional radiomics from the whole tumor and data-driven metabolic subregions, deep models trained on standardized 3D tumor volumes, and a compact intratumoral heterogeneity (ITH) metric from the maximum-uptake slice, then fused these streams at the feature and decision level. The overall framework of the research is given in Figure 1.

## 2. Materials and Methods

### 2.1. Study Samples

This retrospective study was approved by the Institutional Ethics Committee of Fudan University Shanghai Cancer Center (protocol code: SCHBCC-SOAPET, 5 August 2019). A total of 147 eligible patients were included from two cohorts: a primary cohort (n = 129; September 2019–March 2024) and a temporally separated validation cohort (n = 18; April 2024–March 2025).

The inclusion criteria were as follows: (i) primary breast cancer diagnosed by pathological biopsy prior to treatment; (ii) complete clinical and pathological information before and after NAC; (iii) receipt of a complete and standardized course of neoadjuvant therapy without any prior treatment before NAC; and (iv) molecular subtype classified as HER2+. Exclusion criteria included multifocal or bilateral disease, distant metastases, incomplete NAC, or missing clinicopathological data or D-PET images. All enrolled patients received neoadjuvant therapy prior to surgery. Regimens included TCbHP (Docetaxel [T]), or PCbHP (Paclitaxel [P]), in combination with Carboplatin (Cb), Trastuzumab (H), or Pertuzumab (P) for targeted therapy, typically administered over 6 cycles.

The study endpoint was total pCR, defined as ypT0/is and ypN0 on postoperative pathology. Breast and lymph pCR were both recorded; total pCR was coded as “1” only when pCR was achieved in both the breast and axillary lymph nodes; otherwise, it was coded as “0”. We divided the primary cohort into the training set (n = 90) and test set 1 (n = 39) through random sampling (test size = 30%), while the temporally separated cohort (n = 18) was used as an exploratory trend-validation set (test set 2) to assess directional consistency.

### 2.2. Images Acquisition and Tumor Region Segmentation

All patients fasted for at least 4 h prior to imaging. Each patient received an intravenous injection of 110–130 MBq of ^18^F-fluorodeoxyglucose (^18^F-FDG). Blood glucose levels were confirmed to be <10 mmol/L before tracer administration. After a 60 min uptake period under resting conditions, dedicated breast PET imaging was performed using Mammi-PET (Oncovision, Valencia, Spain). The acquisition time was 5–10 min per breast, depending on breast length.

Two experienced nuclear medicine physicians independently drew regions of interest (ROI) using PET Edge software in conjunction with the MIM system (version 6.5.4; MIM Software Inc., Beachwood, OH, USA). Both readers were blinded to all study information except for the laterality of breast cancer. All images were saved in DICOM format, with all direct or indirect personal identifiers removed to ensure patient anonymity.

### 2.3. Baseline Model

To benchmark the incremental value of imaging-derived predictors, we established a baseline model using pre-treatment clinicopathological variables and a conventional PET-derived semiquantitative metric. The model inputs included age, baseline cT stage, baseline cN stage, histological grade, Ki-67 index, neoadjuvant regimen, and SUVmax. In the present cohort, all included HER2-positive patients were estrogen receptor-negative and progesterone receptor-negative; therefore, hormone receptor co-expression was not included as a separate variable in the baseline comparison. Although the predictive value of first-order PET metrics for pCR in HER2-positive breast cancer remains debated and has been reported to be limited in prior studies [24], SUVmax was included because it is widely used, clinically interpretable, and easily obtainable in routine PET assessment. In our cohort, SUVmax had a mean value of 32.20, with a range of 14–65.

A logistic regression classifier was implemented within a scikit-learn pipeline. Continuous variables, including age, Ki-67 index, and SUVmax, were standardized using z-score normalization. Categorical variables (treatment regimen, cT, cN, and histological grade) were encoded via one-hot encoding. Model hyperparameters were optimized in the training set using a grid search with 5-fold stratified cross-validation, maximizing the area under the ROC curve (AUC). Regularization type, regularization strength (C), L1 ratio (for elastic-net), LR solver, and class-weighting (none vs. balanced) were considered.

### 2.4. Image Feature Representation

#### 2.4.1. Radiomics

Images were smoothed with a Gaussian filter to reduce noise. Tumor ROIs were segmented, and K-means clustering (silhouette coefficient, n = 2) was applied to generate two metabolic habitats: a high-uptake region approximating the proliferative tumor core, and a low-uptake region representing necrotic, fibrotic, or stromal components. This habitat imaging framework captures intra-tumoral metabolic heterogeneity, which prior studies have shown to provide complementary prognostic and predictive value [25,26,27].

Radiomic features were extracted for the whole ROI and both subregions using Pyradiomics. A total of 1688-dimensional features were extracted from each region, totaling 5064 dimensions. For feature selection (more details in Appendix A), the Mann–Whitney U test (U-test) was applied, followed by minimum redundancy maximum relevance (mRMR) to further refine the subset. The least absolute shrinkage and selection operator (LASSO) regression was used to determine the final feature set. This yielded five features (three from the whole ROI, two from the high-metabolic region). Model construction compared five machine learning algorithms: logistic regression (LR), random forest (RF), XGBoost (XGB), k-nearest neighbors (KNN), and Gaussian Naive Bayes (GNB). Hyperparameters were optimized using GridSearchCV with stratified five-fold cross-validation on the training set. After selecting the optimal hyperparameter set, each radiomics classifier was refitted on the entire training set to obtain the final model.

#### 2.4.2. Deep Learning

Two architectures were implemented: a 3D ResNet CNN and a 3D ViT transformer. Tumor ROIs with a 5-pixel margin to include the tumor microenvironment were extracted and resampled to 64 × 64 × 64 voxels. The 3D ViT model (https://github.com/lucidrains/vit-pytorch, accessed on 19 April 2025) divided each ROI into non-overlapping patches, projected them to a 64-dimensional latent space, and processed them through 3 transformer layers before a classification head. The 3D ResNet used pretrained r3d_18 weights [28] and consisted of a stem convolution, eight residual blocks, global pooling, and two fully connected layers adapted for binary output.

For deep learning models, we used five-fold cross-validation within the training set for model selection. Architecture comparison and hyperparameter selection were performed using five-fold cross-validation within the training set only. The held-out test sets were not used for model selection, early stopping, learning-rate scheduling, or threshold optimization. Detailed training settings are reported in Appendix A. Candidate training hyperparameter combinations configurations were compared by the mean AUC across the five folds, and the best-performing configuration was selected. Using this selected configuration, a final model was trained. Model performance was primarily evaluated by AUC, with additional metrics including accuracy, sensitivity, and specificity.

#### 2.4.3. ITH

**Figure A2:**
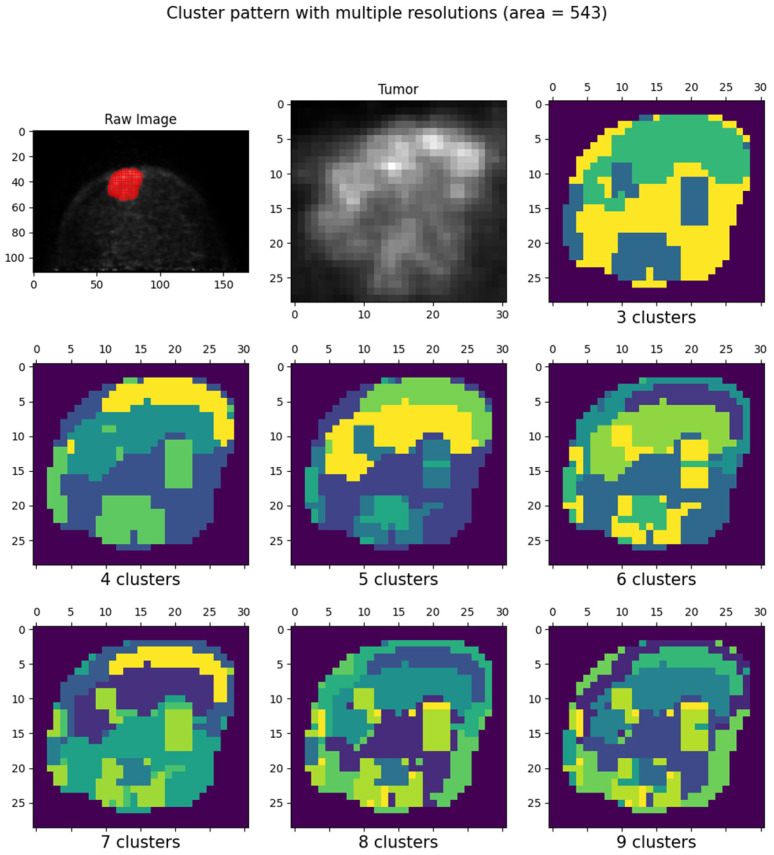
ITH score using cluster patterns derived from D-PET images. The top-left panel shows the original image with the tumor region highlighted in red, and the top-middle panel displays the largest cross-sectional slice of the tumor. Remaining panels depict K-means clustering results (ranging from 3 to 9 clusters) based on per-pixel radiomic features extracted using a 3 × 3 sliding window within the tumor region. Increasing the number of clusters reveals progressively finer spatial heterogeneity patterns, which are subsequently used to calculate the ITH score.

Beyond radiomics and deep learning, we quantified 2D ITH using the ITH score, initially developed for CT [29] and later shown on MRI to predict NAC efficacy in breast cancer [30]. Applied to D-PET, the largest cross-sectional slice of each tumor was analyzed with a 3 × 3 sliding window to extract per-pixel radiomic features, producing a 104-dimensional vector per pixel. K-means clustering assigned pixel clusters, which were mapped back to the image. The ITH score was then calculated from cluster area, connectivity, and spatial distribution, with higher values indicating greater heterogeneity (Figure A2).

### 2.5. Fusion Feature Representation

Recent methodologies on the integration of deep learning and radiomics typically involve combining high-level features extracted from pretrained neural networks with handcrafted radiomic features for downstream machine learning modeling. However, this approach heavily relies on the representation capacity and domain relevance of the pretrained models, which may not generalize well across imaging modalities. Thus, we propose a dual fusion framework that leverages the complementary strengths of radiomics and deep learning, comprising both decision-level fusion and feature-level fusion.

#### 2.5.1. Decision-Level Fusion

Decision-level fusion was performed using a stacking framework. The predicted probabilities from the deep learning model and the radiomics model were used as input variables for a second-level logistic regression model, referred to as the meta-learner. This meta-learner was trained on the training cohort to generate the final fused probability. The hyperparameters of the meta-learner were optimized on the training set, with AUC as the optimization criterion. After finalization, the meta-learner was applied unchanged to the predicted probabilities generated for the test set 1 and test set 2. Additional details are provided in Appendix A.

#### 2.5.2. Feature-Level Fusion

For feature-level fusion, the five radiomics features selected from the final radiomics model were first aligned to the deep feature space at the global pooling layer of the 3D ResNet by a learnable linear projection to the same embedding dimension. The aligned radiomics embedding was then fused with the pooled network representation via element-wise residual addition, yielding an end-to-end trainable feature-level fusion network. The extended network was retrained end-to-end using the same training procedures as the original deep learning model.

### 2.6. Visualization and Interpretability of the Model

In this study, the decision-making process of the deep learning model was visualized using Gradient-weighted Class Activation Mapping (Grad-CAM), intuitively demonstrating the regions of the D-PET images that the deep convolutional neural network focused on during prediction. Radiomics interpretability was assessed using SHAP-based feature attribution and Pearson correlation analysis among the selected features. Fusion-level interpretability was further examined using SHAP analysis of the decision-level fusion model. These approaches addressed the ‘black box’ challenges commonly associated with artificial intelligence by providing enhanced interpretability for both the deep learning and radiomics components of the model.

## 3. Results

### 3.1. Baseline Characteristics of D-PET Cohorts

All patients in this study belonged to the HER2-positive subtype. Baseline clinicopathological characteristics in Table 1 (including age, baseline cT and cN stage, histological grade, Ki-67 index, and neoadjuvant regimen) were comparable between the training set and test sets, with no significant differences (all *p*-values > 0.05). To quantify their combined predictive performance and to benchmark the incremental value of imaging-based models, we constructed a multivariable baseline model using the pre-treatment clinical variables (see Methods; results in Table 2).

The validation strategy was predefined as follows: the test set 1 (n = 39) was used as the primary evaluation cohort, while the test set 2 (n = 18) served as trend-validation cohort. We emphasized uncertainty-aware reporting using bootstrap 95% confidence intervals and interpreted findings in this cohort as directional consistency rather than confirmatory evidence.

### 3.2. Model Performance of Baseline, DL, Radiomics and ITH

Model performance on the training and test set 1 is summarized in Table 2; we used a fixed probability cutoff of 0.5. Overall, the baseline LR model showed limited discriminative ability, achieving an AUC of 0.66 in the training set and an AUC of 0.61 on test set 1. For deep learning, the 3D ResNet model achieved superior results (AUC = 0.79), substantially outperforming the 3D ViT (AUC = 0.72) on test set 1. Among the radiomics classifiers, LR achieved the best performance on test set 1 (AUC = 0.78), with RF and GNB ranking next. By contrast, KNN and XGB exhibited markedly lower performance, possibly reflecting susceptibility to class imbalance and high feature dimensionality.

The ITH score showed no significant association with pCR in the training cohort (*p*-value = 0.23) and only modest predictive value (AUC = 0.61) in logistic regression. Given its modest performance, ITH was excluded from subsequent fusion analyses.

### 3.3. Model Performance of Fusion Models

Table 3 summarizes model performance on the two test sets. Both deep learning and radiomics models showed high sensitivity but only moderate specificity on test set 1, indicating a tendency to minimize false negatives at the cost of some false positives. The 3D ResNet model maintained stable performance on test set 2 (AUC = 0.80).

**Figure 2:**
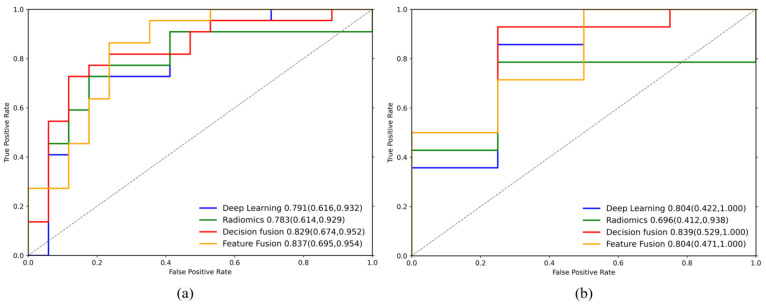
AUC performance on test set 1 (**a**) and test set 2 (**b**).

Both fusion strategies improved overall discrimination relative to the single-branch models. On test set 1, feature-level fusion achieved the highest AUC (0.84), while decision-level fusion (stacking logistic regression) achieved an AUC of 0.83 (Figure 2). At the default cutoff of 0.5, feature-level fusion showed the most balanced threshold-dependent performance on test set 1, with ACC = 0.79, SEN = 0.91, SPE = 0.65, PPV = 0.77, and NPV = 0.85. Decision-level fusion also performed competitively, with ACC = 0.74, SEN = 0.91, SPE = 0.53, PPV = 0.71, and NPV = 0.82.

On test set 2, decision-level fusion achieved the highest AUC (0.84), ACC (0.83), SEN (0.93), and NPV (0.67), whereas radiomics showed the highest SPE (0.75) and PPV (0.91). Given the small and imbalanced size of test set 2, these findings should be interpreted as trend-level validation rather than definitive evidence.

To further interpret the clinical implications of false positives and false negatives, we report PPV and NPV in Table 3. On test set 1, the two fusion models achieved PPV values of 0.71–0.77 and NPV values of 0.82–0.85. On test set 2, PPV/NPV estimates showed wider uncertainty because of the small and imbalanced sample size and should therefore be interpreted cautiously.

**Figure 3:**
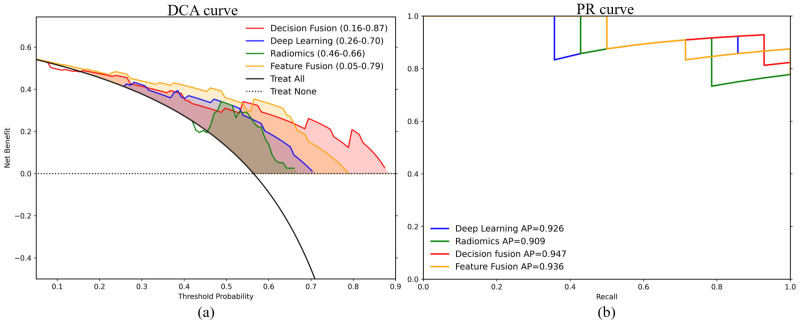
Decision curve analysis for test set 1 (**a**) and precision-recall curves for the class-imbalanced test set 2 (**b**). Probability-threshold intervals in the DCA legend indicate where net benefit exceeds both the treat-all and treat-none strategies. PR curve legends report average precision (AP) values for each model.

Under a two-sided significance level of α = 0.05, we estimated AUC variance using the Hanley–McNeil method and quantified the statistical power for testing AUC > 0.5 in the current test cohorts. In test set 1 (n = 39), all four models achieved statistical power >95%. In contrast, in test set 2 (n = 18), power was markedly constrained by the small sample size and class imbalance, with an overall level of approximately 77%, indicating an increased risk of underpowered inference and limited robustness of negative findings; therefore, these results should be interpreted cautiously. Class imbalance in test set 2 affected the DCA curve, where the ‘treat all’ strategy inflated net benefit. Thus, we plotted precision-recall (PR) curves (Figure 3), which showed that fusion models demonstrated superior performance in terms of precision and recall, with more stable results at higher recall levels.

**Figure 4:**
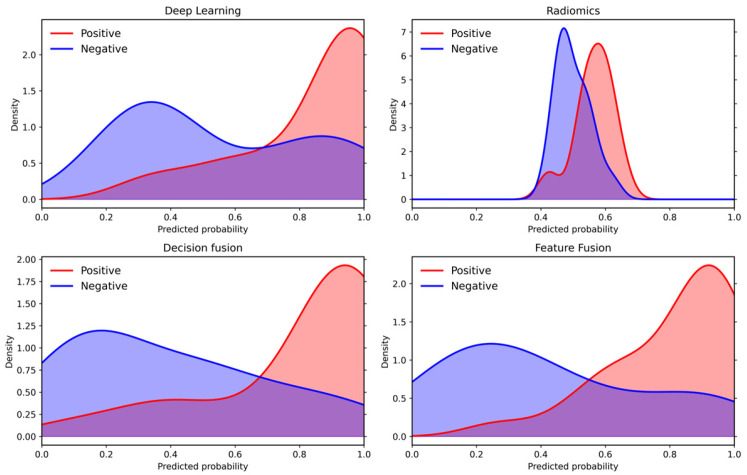
Predicted probability distributions and class separability across models. Density curves for the positive class (Label = 1, red) and the negative class (Label = 0, blue) are shown for the deep learning model, the radiomics model, the decision-level fusion model, and the feature-level fusion model.

DCA on test set 1 showed that the fusion models provided clinical net benefit across broader threshold-probability ranges than the single models. According to the plotted threshold intervals, decision fusion was beneficial over the final continuous range of 0.16–0.87, compared with 0.26–0.70 for deep learning, 0.46–0.66 for radiomics, and 0.05–0.79 for feature fusion. To further examine model behavior, we visualized the distribution of predicted probabilities on test set 1 (Figure 4). The radiomics model showed substantial overlap between positive and negative cases, whereas decision-level fusion reduced the overlap and concentrated more positive cases in the higher-probability range. Feature-level fusion showed variable probability separation across test sets, consistent with the performance fluctuations observed in Table 3.

**Figure A4:**
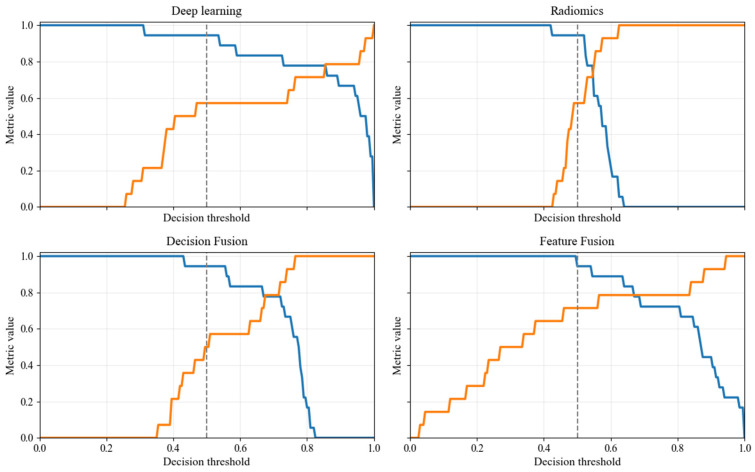
Sensitivity (blue) and specificity (orange) are shown across decision thresholds (0–1). The dashed vertical line marks threshold = 0.5. The curves are provided to support clinically interpretable threshold selection by illustrating trade-offs between false negatives and false positives.

To explicitly support clinical implementation, we additionally performed threshold-response analysis in the primary evaluation cohort (test set 1). Sensitivity and specificity were plotted across the full threshold range for all models (Figure A4), demonstrating the expected trade-off between false negatives and false positives. Clinically, lower thresholds may be preferred when minimizing missed high-risk patients is the priority (e.g., escalation to closer follow-up, multidisciplinary review, or intensified treatment consideration), whereas higher thresholds may be preferred when reducing false-positive-driven overtreatment is prioritized. Thus, the models are intended to support risk stratification and treatment-intensity discussions under different clinical objectives, rather than to impose a single universal cutoff.

### 3.4. Model Explainability Analysis

To systematically evaluate model interpretability, we performed a complementary multi-level analysis incorporating branch-level and fusion-level explanations.

**Figure 5:**
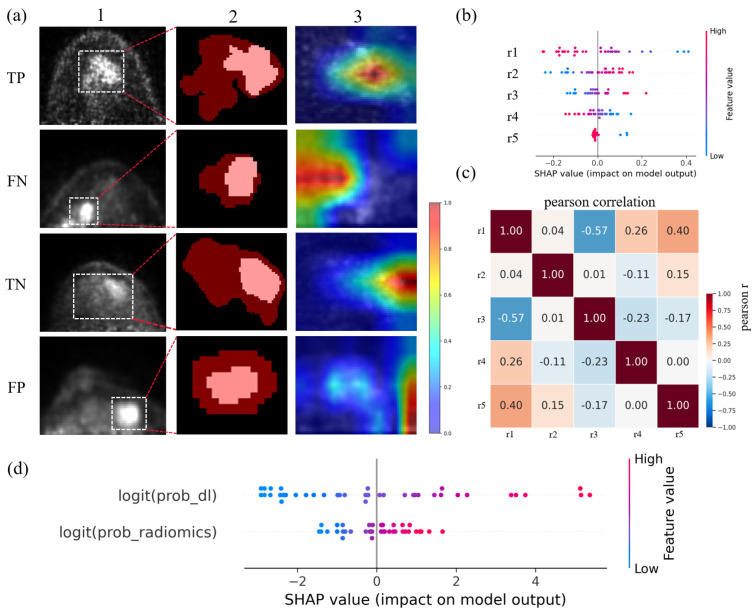
Multidimensional visualization of model decision process and feature contributions. (**a**) Case-level visual explanations for four representative samples. For each case, column 1 shows the D-PET slice with zoomed ROI, column 2 shows the ROI mask with the high-metabolic subregion highlighted, and column 3 displays the Grad-CAM heatmap indicating model attention. Higher heatmap intensity indicates greater relevance (color bar: 0–1). (**b**) SHAP summary plot for the logistic regression radiomics model. SHAP values indicate the direction and magnitude of each selected radiomic feature’s contribution to the model output. (**c**) Pearson correlation heatmap of the five selected radiomic features. Feature information for r1–r5 is provided in Table A1. (**d**) SHAP summary plot for the decision-level fusion model. The two fusion inputs represent the transformed outputs of the deep-learning and radiomics branches, respectively; SHAP values quantify their relative contributions to the final fusion prediction.

For the radiomics branch, Figure 5b presents SHAP-based feature attribution for the logistic-regression model using the five selected radiomic features (r1–r5; selected by U-test, mRMR, and LASSO; Table A1), including two wavelet first-order features, two wavelet texture features, and one LoG-filtered GLCM feature. A bootstrap-based feature-selection stability analysis showed variable-to-moderate selection frequencies for these retained features, ranging from 0.36 to 0.68 (Table A1), supporting their recurrent but exploratory nature. The SHAP summary plot shows the distribution, direction, and magnitude of feature contributions at the sample level. Figure 5c further shows the Pearson correlation matrix of r1–r5, with overall low-to-moderate inter-feature correlations, supporting limited redundancy and complementary information among the selected radiomic descriptors.

To further improve interpretability at the fusion level, we additionally analyzed the decision-level fusion model using SHAP, as shown in Figure 5d. Because the fusion model takes two transformed branch outputs as inputs, the SHAP summary plot directly quantifies the relative contribution of the deep-learning branch and the radiomics branch to the final fused prediction. The plot suggests that both branches contributed to model decisions, while the deep-learning branch tended to show a broader contribution range across samples, indicating greater influence in some cases; meanwhile, the radiomics branch provided complementary information that helped stabilize or refine the final prediction. Together, these results provide supportive visual and feature-attribution evidence for model behavior at the image-attention, handcrafted-feature, and fusion-decision levels.

**Figure 6:**
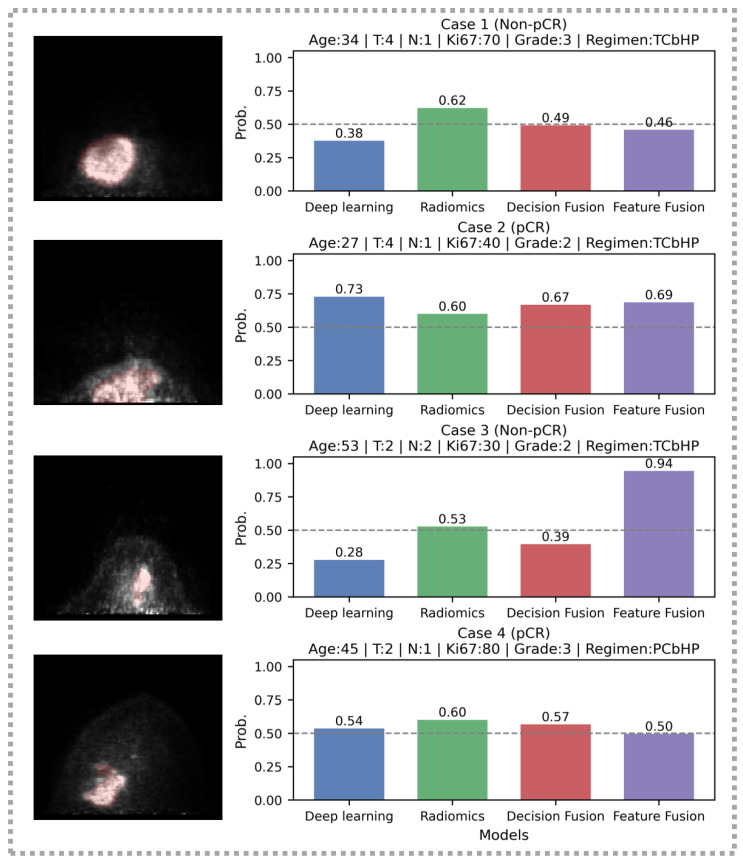
Case-level comparison of four models (deep learning, radiomics, decision fusion, and feature fusion). For each representative case, PET ROI, key clinical background and model-predicted probabilities are shown to illustrate agreement/discordance patterns and the effect of fusion strategies.

To complement the model-level interpretation in Figure 5, we further performed a case-level comparison across all four models in Figure 6, jointly presenting PET regions of interest, clinical context, and predicted probabilities. First, decision-level fusion of deep learning and radiomics exhibited a calibration-like behavior in discordant cases, where one branch predicted positive and the other predicted negative: it often generated intermediate probabilities that moderated extreme single-branch outputs and corrected some branch-specific errors. This case-level behavior is consistent with the fusion-level SHAP analysis in Figure 5d, which showed that the final fusion output was jointly driven by both branch probabilities rather than dominated by a single source in all samples. Second, feature-level fusion demonstrated representational value, yielding correct predictions in three cases; however, in the misclassified case, it still produced a relatively extreme output, suggesting that although feature fusion improves robustness in many scenarios, residual errors remain. Overall, these analyses provide complementary evidence for the interpretability of the multi-model framework and offer useful clues for further optimization and external validation.

## 4. Discussion

In this study, we developed prediction models based on D-PET images to assess the response to NAC from three perspectives: radiomics, deep learning, and quantitative heterogeneity. Both the deep learning and radiomics models showed good discriminative ability on test set 1, achieving AUCs of 0.79 and 0.78, respectively, supporting the potential value of baseline D-PET imaging in predicting tumor response. Fusion models further improved performance. On the primary test set, feature-level fusion achieved the highest AUC (0.84), while decision-level fusion also performed strongly (AUC 0.83). On the independent trend-validation set, decision-level fusion achieved the highest AUC (0.84), whereas feature-level fusion showed a slightly lower but still competitive AUC (0.80).

Importantly, we deliberately focused on single-timepoint, pre-NAC D-PET to answer a pre-treatment decision question; serial imaging is often impractical in routine care, especially for D-PET given cost and access constraints. Prior work also shows that early baseline prediction can enable proactive treatment decisions [31]. In the traditional-modality literature, multi-timepoint or multimodal designs often perform better [23,32,33]. However, when restricted to baseline-only images, performance is typically moderate. By contrast, D-PET distinctly offers volumetric, high-resolution functional imaging of the breast without compression or respiratory motion artifacts. Prone positioning and a breast-dedicated acquisition mitigate partial-volume effects and enable accurate quantification of local metabolic activity, which is crucial for detecting therapeutic response before morphologic changes are apparent [34,35,36]. These properties likely contribute to the competitive performance achieved using baseline-only D-PET in our cohort.

From a clinical perspective, the baseline model demonstrated limited discrimination (Table 2), suggesting that conventional pre-treatment clinical indicators alone may be insufficient to reliably predict pCR in this HER2-positive cohort. This observation is in line with prior reports that, in HER2-positive patients receiving dual-targeted therapy (e.g., trastuzumab plus pertuzumab), traditional clinicopathological factors can be less predictive of pCR, potentially due to the high efficacy of systemic therapy and reduced variability in response drivers across patients [37].

Beyond systemic treatment stratification, pCR prediction may also have important implications for surgical planning in HER2-positive breast cancer. Neoadjuvant therapy is expected to increase the feasibility of breast-conserving surgery, particularly in patients with substantial tumor response; however, conversion from initially planned mastectomy to breast-conserving surgery is not always achieved in routine practice. A recent retrospective study of patients with complete remission of the primary tumor after neoadjuvant therapy showed that baseline tumor extension and focality were major predictors of mastectomy, while breast-conserving surgery did not negatively affect survival outcomes [38]. These findings highlight the importance of carefully reassessing surgical indications after treatment response and avoiding potentially unnecessary mastectomy in selected patients. In this context, a reliable noninvasive model for pCR prediction may support multidisciplinary surgical planning by identifying patients who warrant closer reassessment for breast-conserving approaches, while also considering residual imaging findings, tumor-to-breast ratio, focality, patient preference, and oncological safety. Importantly, such models should complement, rather than replace, standard post-NAC imaging, surgical judgment, and pathological confirmation.

Deep learning and radiomics provided complementary strengths. The 3D ResNet outperformed the 3D ViT on test set 1, which is consistent with the practical advantage of CNN-based architectures in modest datasets and in settings where image resolution and sample size may limit transformer performance. Radiomics, in contrast, distilled multiscale intensity and texture statistics that remain sample-efficient and interpretable. We hypothesize that fusion can reduce model-specific blind spots by combining these representations. In decision-level fusion, a lightweight stacking logistic-regression meta-learner was used to integrate the deep-learning and radiomics probabilities. Under the current sample size, this simple meta-learner provided competitive and stable performance while remaining easy to interpret. More complex decision-level fusion schemes may still be worth exploring in larger cohorts.

Fusion models appeared to improve performance in this exploratory analysis. On the primary test set, feature-level fusion achieved the highest AUC (0.84), while decision-level fusion also performed strongly (AUC 0.83). On the independent trend-validation set, decision-level fusion achieved the highest AUC (0.84), whereas feature-level fusion showed a slightly lower but still competitive AUC (0.80). On test set 1, the fusion models yielded PPV values of 0.71–0.77 and NPV values of 0.82–0.85, suggesting that fusion improved overall discrimination while maintaining clinically meaningful threshold-dependent performance. However, because test set 2 was small and imbalanced, the apparent advantages observed there should be interpreted cautiously. In practice, the model should be viewed as a decision-support tool to complement multidisciplinary assessment rather than a standalone determinant of therapy, and operating thresholds should be selected based on toxicity profiles, alternative treatment options, patient comorbidities, and patient preferences.

Model interpretability provided useful but exploratory insights into model behavior and helped contextualize the fusion results. Radiomics predictors were predominantly wavelet-derived features capturing multiscale intensity and texture patterns. Both whole-tumor and high-uptake subregion descriptors contributed to the radiomics model, and Grad-CAM often highlighted regions overlapping with relatively high FDG uptake (Figure 5). However, these observations should be interpreted as supportive and hypothesis-generating rather than confirmatory evidence of biological validity. Grad-CAM and SHAP analyses do not establish causal mechanisms or prove that the highlighted regions represent biologically distinct tumor habitats. Future studies should validate these interpretability patterns through correlation with histopathology, molecular markers, or spatially resolved biological data. In addition, although our study focused on intra-tumoral heterogeneity, recent evidence suggests peritumoral features are also prognostically meaningful. Incorporating peritumoral and microenvironment features may further enhance model performance [39,40,41].

Several methodological aspects merit discussion. First, transfer learning for D-PET remains challenging due to domain mismatch. Widely used backbones pretrained on natural videos or on CT and MRI carry inductive biases and distribution gaps for D-PET. In our experiments, r3d_18 weights [42] pretrained on large-scale videos outperformed Medical Net [43] pretrained on medical images, with AUCs of 0.79 and 0.75, respectively, suggesting that spatiotemporal biases learned from videos can transfer to heterogeneous D-PET signals despite cross-domain shift. This provides a practical interim strategy but also highlights the need for D-PET-specific pretraining resources.

Second, the exploratory intra-tumoral heterogeneity model performed only modestly on D-PET, with an AUC of 0.61, which likely reflects a methodological mismatch because a two-dimensional, single-slice implementation was applied to inherently three-dimensional, multi-scale metabolic patterns. Restricting analysis to the largest slice discards volumetric organization such as core–rim structure, superior–inferior gradients, and multifocal hotspots, and it amplifies partial-volume and spillover effects. A fixed three-by-three window with k-means, operating near D-PET’s effective resolution, produces noise-sensitive clusters that miss larger habitats. Combined with D-PET’s lower anatomical sharpness, boundary-dependent two-dimension textures become unstable. To exploit D-PET’s strengths, future work should adopt genuinely three-dimensional, multi-scale heterogeneity metrics and scale adaptive clustering with denoising and intensity normalization to enable robust volumetric identification of high-uptake subregions that are most relevant for response prediction, which would align with our Grad-CAM observations.

Third, we did not comprehensively benchmark alternative fusion architectures. Future work should systematically compare early feature-level concatenation, late probability-level weighting, and hybrid attention-based fusion, alongside calibration and net-benefit analyses to support clinical translation. In addition, we note that the landscape of medical AI is rapidly evolving, including large language models (LLMs) and multimodal foundation models being explored for oncology tasks and treatment–response assessment across cancer types [44,45]. Although LLM-centric methods are not the focus of the current D-PET-based study, these developments highlight promising directions for future work, such as integrating structured clinical text, harmonizing multimodal information, and enabling more flexible decision-support systems under limited sample sizes.

Despite these promising findings, several limitations should be acknowledged. First, this is an exploratory, hypothesis-generating study, and the proposed model should be viewed primarily as a decision-support tool rather than a stand-alone diagnostic solution. The dataset is moderate in size and derived from a single center, reflecting the cost and limited accessibility of D-PET. Test set 1 was generated by random splitting of the primary same-institution cohort and therefore represents an internal holdout set rather than external validation. Although the test set 2 was temporally separated, it was still derived from the same institution and contained only 18 patients; therefore, it provides only preliminary evidence of temporal consistency rather than confirmation of external generalizability. Its small and imbalanced composition also resulted in wide uncertainty for several performance estimates and limited statistical power.

Second, the study population was relatively selected. The exclusion of patients with multifocal or bilateral disease, distant metastases, incomplete neoadjuvant treatment, or missing imaging/clinical data helped ensure reliable lesion-level modeling, standardized response labeling, and consistent model development. However, these criteria also created a more idealized cohort than that encountered in routine clinical practice, where patients may present with more complex disease patterns, variable treatment completion, and incomplete data availability. Moreover, the relatively uniform anti-HER2 neoadjuvant regimens improved treatment homogeneity but may limit applicability to patients treated with different regimens across institutions. Larger prospective, multi-institutional external validation cohorts with broader disease presentations, more diverse treatment strategies, and harmonized D-PET acquisition and preprocessing protocols are required before the robustness, transportability, and clinical utility of the proposed models can be established.

Third, the number of patients remains modest relative to the complexity of the modeling framework, which included radiomics feature selection, deep-learning model development, and two fusion strategies. Although all feature-selection and model-tuning procedures were restricted to the training set, residual model-selection bias and overfitting cannot be fully excluded. The current fusion strategy emphasized simplicity and robustness under limited sample size; however, more advanced fusion architectures may capture richer cross-representation interactions and should be evaluated in larger datasets with rigorous nested validation and truly external testing. In addition, although we assessed discrimination, calibration, and decision-analytic performance, these analyses remain preliminary because of the limited cohort size and class imbalance. Additional prospective, workflow-oriented validation is needed to determine whether the proposed framework can improve real-world multidisciplinary decision-making, and future work should also evaluate its applicability to broader molecular subtypes.

## 5. Conclusions

In this exploratory subtype-specific D-PET study of HER2+ breast cancer, baseline D-PET showed promising potential for noninvasive prediction of pCR after NAC. The fusion of deep learning and conventional radiomics was associated with more balanced performance than either approach alone. Larger prospective multi-center studies are needed to confirm model robustness, generalizability, and clinical utility.

## Figures and Tables

**Table 1:** Comparison of clinical characteristics between the training and test sets.

Characteristics	Training Set (n = 90)	Test Set 1 (n = 39)	Test Set 2 (n = 18)	*p* Value
Age, median (range)	52 (26–69)	57 (31–71)	51 (34–62)	0.14
Ki-67 index				0.32
≤20%	8 (8.9%)	7 (17.9%)	2 (11.1%)	
>20%	82 (91.1%)	32 (82.1%)	16 (88.9%)	
TNM staging (tumor)				
T1	2 (2.2%)	1 (2.6%)	1 (5.6%)	
T2	60 (66.7%)	19 (48.7%)	14 (77.8%)	
T3	9 (10.0%)	5 (12.8%)	1 (5.6%)	
T4	19 (21.1%)	14 (35.9%)	2 (11.1%)	
TNM staging (nodus)				0.25
N1	54 (60.0%)	22 (56.4%)	9 (50.0%)	
N2	12 (13.3%)	9 (23.1%)	6 (33.3%)	
N3	16 (17.7%)	5 (12.8%)	2 (11.1%)	
Histological grade				0.42
2	34 (37.8%)	12 (30.8%)	9 (50.0%)	
3	56 (62.2%)	27 (69.2%)	9 (50.0%)	
Therapeutic regimen (%)				0.33
PCbHP	30 (33.3%)	11 (28.2%)	4 (22.2%)	
TCbHP	60 (66.7%)	28 (71.8%)	14 (77.8%)	
location (left) (%)	44 (48.9%)	19 (48.7%)	13 (72.2%)	0.48
Breast pCR (%)	60 (66.7%)	27 (69.2%)	15 (83.3%)	0.29
Lymph pCR (%)	75 (83.3%)	33 (84.6%)	15 (83.3%)	0.96
Total pCR (%)	59 (65.5%)	22 (56.4%)	14 (77.8%)	0.30

Note. Continuous variables are presented as median (range) and were compared across the three data sets using the Kruskal–Wallis test. Categorical variables are presented as a number (percentage) and were compared using the chi-square test or Fisher exact test, as appropriate. *p* values represent overall comparisons across all data sets.

**Table 2:** Model performance comparison.

Methods	Models	Training Set (n = 90)				Test Set 1 (n = 39)			
		AUC	ACC	SEN	SPE	AUC	ACC	SEN	SPE
Deep learning	3D ViT	0.95 (0.91,1.00)	0.86 (0.76,0.98)	0.98 (0.94,1.00)	0.61 (0.45,0.73)	0.72 (0.60,0.88)	0.72 (0.61,0.78)	0.91 (0.73,1.00)	0.47 (0.20,0.69)
	3D Resnet	0.93 (0.86,0.98)	0.87 (0.79,0.93)	0.97 (0.91,1.00)	0.68 (0.50,0.83)	0.79 (0.61,0.94)	0.79 (0.63,0.91)	0.95 (0.80,1.00)	0.59 (0.28,0.82)
Radiomics	RF	0.88 (0.84,0.93)	0.78 (0.69,0.86)	0.95 (0.89,1.00)	0.45 (0.28,0.63)	0.75 (0.70,0.81)	0.74 (0.56,0.88)	0.95 (0.82,1.00)	0.47 (0.18,0.71)
	XGB	0.86 (0.79,0.93)	0.74 (0.64,0.83)	0.97 (0.91,1.00)	0.32 (0.17,0.48)	0.70 (0.50,0.88)	0.67 (0.57,0.84)	0.91 (0.82,1.00)	0.35 (0.11,0.64)
	GNB	0.74 (0.63,0.83)	0.70 (0.60,0.79)	0.85 (0.75,0.93)	0.42 (0.24,0.59)	0.77 (0.60,0.95)	0.77 (0.63,0.88)	0.95 (0.82,1.00)	0.53 (0.23,0.77)
	KNN	0.70 (0.61,0.79)	0.70 (0.58,0.81)	0.92 (0.84,0.98)	0.29 (0.14,0.45)	0.62 (0.43,0.82)	0.62 (0.41,0.72)	0.95 (0.74,1.00)	0.18 (0.00,0.33)
	LR	0.74 (0.64,0.82)	0.74 (0.62,0.83)	0.90 (0.82,0.98)	0.45 (0.38,0.58)	0.78 (0.61,0.93)	0.79 (0.64,0.90)	0.91 (0.77,1.00)	0.65 (0.45,0.82)
Baseline	LR	0.66 (0.54,0.78)	0.61 (0.51,0.71)	0.66 (0.54,0.78)	0.52 (0.35,0.68)	0.61 (0.41,0.81)	0.62 (0.46,0.77)	0.59 (0.39,0.77)	0.65 (0.41,0.88)

Notes. 3D ViT, three-dimensional vision transformer; SEN, sensitivity; SPE, specificity; ACC, accuracy; CI, confidence interval. Performance metrics are reported as actual values accompanied by 95% CI.

**Table 3:** Comparison of predictive performance for deep learning, radiomics, and fusion models on two test sets.

Methods	Test Set 1 (n = 39)						Test Set 2 (n = 18)					
	AUC	ACC	SEN	SPE	PPV	NPV	AUC	ACC	SEN	SPE	PPV	NPV
Deep learning	0.79 (0.62,0.93)	0.79 (0.63,0.91)	0.95 (0.80,1.00)	0.59 (0.28,0.82)	0.75 (0.54,0.91)	**0.91** **(0.62,1.00)**	0.80 (0.42,1.00)	0.78 (0.55,0.94)	0.86 (0.67,1.00)	0.50 (0.00,1.00)	0.86 (0.64,1.00)	0.50 (0.00,1.00)
Radiomics	0.78 (0.61,0.93)	0.77 (0.64,0.90)	0.91 (0.77,1.00)	0.59 (0.35,0.82)	0.74 (0.55,0.91)	0.83 (0.62,1.00)	0.70 (0.41,0.94)	0.72 (0.50,0.89)	0.71 (0.47,0.93)	**0.75** **(0.00,1.00)**	**0.91** **(0.69,1.00)**	0.43 (0.00,0.83)
Decision fusion	0.83 (0.67,0.95)	0.74 (0.62,0.87)	**0.91** **(0.81,1.00)**	0.53 (0.42,0.75)	0.71 (0.54,0.88)	0.82 (0.55,1.00)	**0.84** **(0.53,1.00)**	**0.83** **(0.67,1.00)**	**0.93** **(0.77,1.00)**	0.50 (0.00,1.00)	0.87 (0.67,1.00)	**0.67** **(0.00,1.00)**
Feature fusion	**0.84** **(0.69,0.95)**	**0.79** **(0.67,0.92)**	0.91 (0.77,1.00)	**0.65** **(0.40,0.88)**	**0.77** **(0.63,0.95)**	0.85 (0.70,1.00)	0.80 (0.47,1.00)	0.72 (0.50,0.89)	0.79 (0.54,1.00)	0.50 (0.00,1.00)	0.85 (0.62,1.00)	0.40 (0.00,1.00)

Notes. Boldface indicates the highest value for each metric in each dataset. Performance metrics are reported as actual values accompanied by 95% CI.

**Figure A1:**
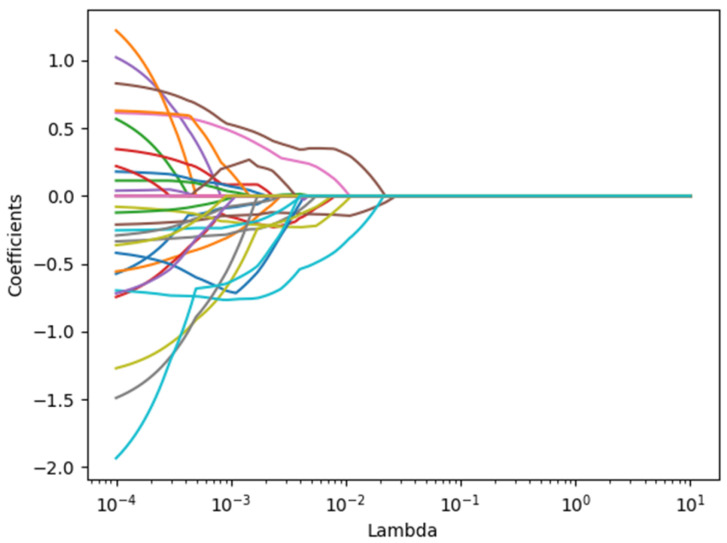
LASSO coefficient profiles as a function of the regularization parameter lambda. Each colored curve represents the coefficient trajectory of one candidate radiomic feature as lambda changes. As lambda increases, more feature coefficients are shrunk toward zero, enabling selection of the most informative features through penalization.

**Figure A3:**
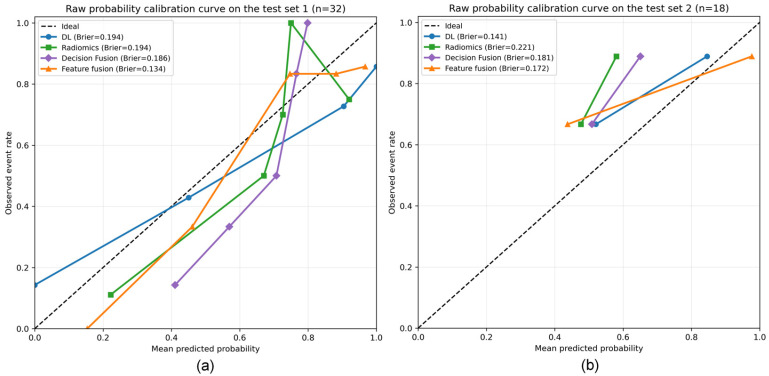
Calibration curves of the primary models on the two held-out test sets. (**a**) Test set 1; (**b**) test set 2. Reliability diagrams are shown for deep learning (DL), radiomics, decision-level fusion, and feature-level fusion using the raw predicted probabilities.

## Data Availability

All original code used for preprocessing, model training, inference, and statistical evaluation has been deposited on the GitHub (https://github.com/zength123/NAC-predict, accessed on 29 April 2026) and is publicly available. The repository includes model weights, intermediate analysis outputs, and usage documentation to facilitate reproducibility. The radiology images and clinical data are available from the lead contact, Jingyi Cheng (Email: fudansphic-nm@outlook.com) and Wenlong Ming (Email: wming@nuist.edu.cn), upon reasonable request.

## Abbreviations

The following abbreviations are used in this manuscript:

D-PET	Dedicated Breast Positron Emission Tomography
HER2	Human Epidermal Growth Factor Receptor 2
pCR	Pathological Complete Response
NAC	Neoadjuvant Chemotherapy
DL	Deep Learning
ITH	Intratumoral Heterogeneity
ROI	Region of Interest
FDG	Fluorodeoxyglucose
CNN	Convolutional Neural Network
ViT	Vision Transformer
PPV	Positive Predictive Value
NPV	Negative Predictive Value
LR	Logistic Regression
RF	Random Forest
XGB	XGBoost
GNB	Gaussian Naive Bayes
KNN	K-Nearest Neighbors

## Appendix A

To ensure strict separation between model development and final evaluation, all parameter estimation, feature selection, hyperparameter tuning, and fusion-model training were performed using the training set only. Test set 1 and test set 2 were not used in any step of model development and were reserved exclusively for final evaluation.

(1) Radiomic features were extracted from the following image types:

Original images;

Wavelet-transformed images;

Logarithm-transformed images;

Gradient images;

Square images;

Square root images;

Exponential images;

Laplacian of Gaussian (LoG) filtered images with sigma = 2.0, 3.0, 4.0, and 5.0.

(2) The feature classes included:

First-order statistics;

Shape features;

Gray Level Co-occurrence Matrix (GLCM);

Gray Level Run Length Matrix (GLRLM);

Gray Level Size Zone Matrix (GLSZM);

Gray Level Dependence Matrix (GLDM);

Neighboring Gray Tone Difference Matrix (NGTDM).

(3) Feature selection was conducted using the training set only to avoid information leakage. A three-step procedure was applied:

Step 1: Univariate screening. Each radiomic feature was screened using a two-sided Mann–Whitney U tests. Features with *p* < 0.05 were retained as candidates for multivariable modeling (retained: n = 123). No multiple-comparison correction (e.g., FDR) was applied at this stage because this test was used only as a permissive, initial dimensionality-reduction step (pre-filtering) prior to subsequent redundancy control and regularized modeling, rather than for making inferential claims about individual features.

Step 2: Minimum redundancy maximum relevance (mRMR). After univariate screening, mRMR was used to reduce feature redundancy while preserving relevance to pCR. We employed the MIQ (Mutual Information Quotient) criterion as implemented in the ‘pymrmr’ package and retained the top 30 ranked features. The number of features retained at the mRMR stage was pre-specified as a compromise between retaining sufficient candidate information and controlling the dimensionality before LASSO-based sparse selection.

Step 3: LASSO. LASSO was used as an embedded feature-selection step to obtain a sparse feature subset and further mitigate overfitting. Candidate regularization parameters (λ) were sampled on a log-spaced grid ranging from 1 × 10^−4^ to 1 × 10 (2000 values). The optimal λ was selected using 5-fold cross-validation on the training set, choosing the λ that minimized the mean cross-validated error. After selecting λ, the LASSO model was refitted on the full training set, and features with non-zero coefficients were retained as the final radiomics signature. The resulting selected features were then used as inputs for downstream predictive modeling with multiple machine-learning classifiers.

(4) For the deep learning approaches, two architectures were employed—a convolutional neural network (CNN) based on 3D ResNet and a transformer-based model using 3D ViT—to classify tumor regions of interest (ROIs). The tumor ROIs included a 5-pixel margin to capture the tumor microenvironment. Each ROI was resampled to a fixed size of 64 × 64 × 64 voxels using trilinear interpolation, and intensity values were normalized to the range of [0, 1] via min–max normalization for each ROI. Each model took a single-channel PET image as input. Hyperparameter combinations (including batch size, learning rate, loss criterion, etc.) were selected through five-fold cross-validation on the training data. The final model was then trained on the complete training set using the selected configuration, with 10% of the training data randomly split for validation during training to enable procedures such as early stopping.

The 3D ResNet model was adapted from r3d_18 with kinetics pre-trained weights. The first convolution was modified for single-channel input, and the classification head was replaced with two fully connected layers (512 → 128 → 2) for binary prediction. The model was trained using AdamW with an initial learning rate of 1 × 10^−4^ and cosine annealing scheduling. Focal Loss (α = 1, γ = 2) was used to address class imbalance, and the batch size was 16. The 3D ViT model followed the vit_pytorch.vit_3d implementation, using a patch size of 8, an embedding dimension of 64, three transformer layers, four attention heads, and an MLP hidden dimension of 256. The MLP dropout rate was set to 0.30. The model was optimized with AdamW using a weight decay of 1 × 10^−2^. We used a warmup + cosine learning-rate schedule: the learning rate was linearly warmed up over the first 10% of the total training epochs to a peak learning rate, and then decayed following a cosine schedule from the peak learning rate to a minimum learning rate (2 × 10^−5^) for the remainder of training, and the batch size was 8.

(5) For feature-level fusion, radiomic features were standardized (using the training set mean and variance) and paired with their corresponding images in the dataset according to their unique file identifiers. During network training, the selected radiomic feature vector (dimension 5) was mapped to a 128-dimensional representation via a linear layer, while deep features extracted from the 3D ResNet backbone underwent global average pooling and the same linear mapping. These two representations were integrated via element-wise addition and jointly input into the classification head, enabling the network to learn an optimal combination of handcrafted and automatically extracted features in an end-to-end manner. This design allows the model to leverage complementary information: radiomics encodes quantitative lesion characteristics rooted in domain knowledge, whereas deep learning captures high-level image semantics. The fusion is theoretically justified by the premise that combining handcrafted and learned features expands the feature space and provides both semantic richness and clinical interpretability.

Stacking-LR fusion uses predicted probabilities from both the deep learning model and the radiomics model. The model was trained using the logit-transformed probabilities to avoid numerical instability near the boundaries of 0 and 1. The trained logistic regression model then outputs a fused probability, integrating both sources of information to improve predictive performance.

**Table A1:** The distribution of the feature subsets after selection.

Feature Name	Mapping (r1–r5)	*p*-Value	Coefficients	Frequency	Cluster
wavelet-HLH_firstorder_Range	r3	0.047	0.10	0.36	High
wavelet-HLL_firstorder_Skewness	r4	0.037	−0.11	0.42	High
wavelet-HHL_ngtdm_Contrast	r5	0.035	−0.14	0.44	High
wavelet-HLH_glcm_Imc1	r2	0.002	0.35	0.68	ALL
log-sigma-5-0-mm-3D_glcm_Imc2	r1	0.018	−0.41	0.64	ALL

**Notes.** The cluster column indicates the region from which the features were derived: “ALL” represents features derived from the entire tumor mask region, while “High” refers to features derived from the high metabolic regions obtained after clustering the mask into two categories, namely high and low metabolism. The r1–r5 labels correspond to the features depicted in the figures in the main text. The *p*-values were calculated using the Mann–Whitney U test (*p*-value < 0.05) for preliminary feature selection. Non-zero feature coefficients were selected by LASSO regression and indicate the relative importance and direction, positive or negative association, of each radiomics feature retained in the final model. Selection frequency was calculated using 50 repeated bootstrap resampling iterations within the training cohort. In each iteration, 90% cases were sampled with replacement from the training set, and the complete feature-selection pipeline, including Mann–Whitney U-test screening, mRMR ranking, and LASSO selection, was repeated. Selection frequency represents the proportion of iterations in which each retained feature was selected by the final LASSO step.

## References

[1] Bray F., Laversanne M., Sung H., Ferlay J., Siegel R.L., Soerjomataram I., Jemal A. (2024). Global cancer statistics 2022: GLOBOCAN estimates of incidence and mortality worldwide for 36 cancers in 185 countries. CA Cancer J. Clin..

[2] (2017). Adjuvant Pertuzumab and Trastuzumab in Early HER2-Positive Breast Cancer. N. Engl. J. Med..

[3] Perez E.A., Romond E.H., Suman V.J., Jeong J.H., Sledge G., Geyer C.E., Martino S., Rastogi P., Gralow J., Swain S.M. (2014). Trastuzumab plus adjuvant chemotherapy for human epidermal growth factor receptor 2-positive breast cancer: Planned joint analysis of overall survival from NSABP B-31 and NCCTG N9831. J. Clin. Oncol..

[4] Bradley R., Braybrooke J., Gray R., Hills R., Liu Z., Peto R., Davies L., Dodwell D., McGale P., Pan H. (2021). Trastuzumab for early-stage, HER2-positive breast cancer: A meta-analysis of 13 864 women in seven randomised trials. Lancet Oncol..

[5] Cameron D., Piccart-Gebhart M.J., Gelber R.D., Procter M., Goldhirsch A., de Azambuja E., Castro G., Untch M., Smith I., Gianni L. (2017). 11 years’ follow-up of trastuzumab after adjuvant chemotherapy in HER2-positive early breast cancer: Final analysis of the HERceptin Adjuvant (HERA) trial. Lancet.

[6] Tateishi U., Miyake M., Nagaoka T., Terauchi T., Kubota K., Kinoshita T., Daisaki H., Macapinlac H.A. (2012). Neoadjuvant chemotherapy in breast cancer: Prediction of pathologic response with PET/CT and dynamic contrast-enhanced MR imaging--prospective assessment. Radiology.

[7] Rousseau C., Devillers A., Sagan C., Ferrer L., Bridji B., Campion L., Ricaud M., Bourbouloux E., Doutriaux I., Clouet M. (2006). Monitoring of early response to neoadjuvant chemotherapy in stage II and III breast cancer by [18F]fluorodeoxyglucose positron emission tomography. J. Clin. Oncol..

[8] Sakaguchi R., Kataoka M., Kanao S., Miyake K.K., Nakamoto Y., Sugie T., Toi M., Mikami Y., Togashi K. (2019). Distribution pattern of FDG uptake using ring-type dedicated breast PET in comparison to whole-body PET/CT scanning in invasive breast cancer. Ann. Nucl. Med..

[9] Teixeira S.C., Rebolleda J.F., Koolen B.B., Wesseling J., Jurado R.S., Stokkel M.P., Del Puig Cózar Santiago M., van der Noort V., Rutgers E.J., Valdés Olmos R.A. (2016). Evaluation of a Hanging-Breast PET System for Primary Tumor Visualization in Patients With Stage I-III Breast Cancer: Comparison With Standard PET/CT. AJR Am. J. Roentgenol..

[10] Eisenhauer E.A., Therasse P., Bogaerts J., Schwartz L.H., Sargent D., Ford R., Dancey J., Arbuck S., Gwyther S., Mooney M. (2009). New response evaluation criteria in solid tumours: Revised RECIST guideline (version 1.1). Eur. J. Cancer.

[11] Jannusch K., Dietzel F., Bruckmann N.M., Morawitz J., Boschheidgen M., Minko P., Bittner A.K., Mohrmann S., Quick H.H., Herrmann K. (2024). Prediction of therapy response of breast cancer patients with machine learning based on clinical data and imaging data derived from breast [(18)F]FDG-PET/MRI. Eur. J. Nucl. Med. Mol. Imaging.

[12] Gu J., Tong T., He C., Xu M., Yang X., Tian J., Jiang T., Wang K. (2022). Deep learning radiomics of ultrasonography can predict response to neoadjuvant chemotherapy in breast cancer at an early stage of treatment: A prospective study. Eur. Radiol..

[13] Stefano A. (2024). Challenges and limitations in applying radiomics to PET imaging: Possible opportunities and avenues for research. Comput. Biol. Med..

[14] Lee H.-j., Lee J.H., Lee J.E., Na Y.M., Park M.H., Lee J.S., Lim H.S. (2024). Prediction of early clinical response to neoadjuvant chemotherapy in Triple-negative breast cancer: Incorporating Radiomics through breast MRI. Sci. Rep..

[15] Shi Z., Huang X., Cheng Z., Xu Z., Lin H., Liu C., Chen X., Liu C., Liang C., Lu C. (2023). MRI-based Quantification of Intratumoral Heterogeneity for Predicting Treatment Response to Neoadjuvant Chemotherapy in Breast Cancer. Radiology.

[16] Orlhac F., Boughdad S., Philippe C., Stalla-Bourdillon H., Nioche C., Champion L., Soussan M., Frouin F., Frouin V., Buvat I. (2018). A Postreconstruction Harmonization Method for Multicenter Radiomic Studies in PET. J. Nucl. Med..

[17] Xu Z., Zhou Z., Son J.B., Feng H., Adrada B.E., Moseley T.W., Candelaria R.P., Guirguis M.S., Patel M.M., Whitman G.J. (2025). Deep Learning Models Based on Pretreatment MRI and Clinicopathological Data to Predict Responses to Neoadjuvant Systemic Therapy in Triple-Negative Breast Cancer. Cancers.

[18] Chen Y., Wang L., Dong X., Luo R., Ge Y., Liu H., Zhang Y., Wang D. (2023). Deep Learning Radiomics of Preoperative Breast MRI for Prediction of Axillary Lymph Node Metastasis in Breast Cancer. J. Digit. Imaging.

[19] Luo L., Wang X., Lin Y., Ma X., Tan A., Chan R., Vardhanabhuti V., Chu W.C., Cheng K.T., Chen H. (2025). Deep Learning in Breast Cancer Imaging: A Decade of Progress and Future Directions. IEEE Rev. Biomed. Eng..

[20] Kuzmova M., Cullinane C., Rutherford C., McCartan D., Rothwell J., Evoy D., Geraghty J., Prichard R.S. (2023). The accuracy of MRI in detecting pathological complete response following neoadjuvant chemotherapy in different breast cancer subtypes. Surg. Oncol..

[21] Gampenrieder S.P., Peer A., Weismann C., Meissnitzer M., Rinnerthaler G., Webhofer J., Westphal T., Riedmann M., Meissnitzer T., Egger H. (2019). Radiologic complete response (rCR) in contrast-enhanced magnetic resonance imaging (CE-MRI) after neoadjuvant chemotherapy for early breast cancer predicts recurrence-free survival but not pathologic complete response (pCR). Breast Cancer Res..

[22] Gu Y.L., Pan S.M., Ren J., Yang Z.X., Jiang G.Q. (2017). Role of Magnetic Resonance Imaging in Detection of Pathologic Complete Remission in Breast Cancer Patients Treated With Neoadjuvant Chemotherapy: A Meta-analysis. Clin. Breast Cancer.

[23] Feng X., Shi Y., Wu M., Cui G., Du Y., Yang J., Xu Y., Wang W., Liu F. (2025). Predicting the efficacy of neoadjuvant chemotherapy in breast cancer patients based on ultrasound longitudinal temporal depth network fusion model. Breast Cancer Res..

[24] Oliveira C., Oliveira F., Constantino C., Alves C., Brito M.J., Cardoso F., Costa D.C. (2024). Baseline [(18)F]FDG PET/CT and MRI first-order breast tumor features do not improve pathological complete response prediction to neoadjuvant chemotherapy. Eur. J. Nucl. Med. Mol. Imaging.

[25] Li S., Dai Y., Chen J., Yan F., Yang Y. (2024). MRI-based habitat imaging in cancer treatment: Current technology, applications, and challenges. Cancer Imaging.

[26] Hatt M., Cheze Le Rest C., Antonorsi N., Tixier F., Tankyevych O., Jaouen V., Lucia F., Bourbonne V., Schick U., Badic B. (2021). Radiomics in PET/CT: Current Status and Future AI-Based Evolutions. Semin. Nucl. Med..

[27] Sui C., Su Q., Chen K., Tan R., Wang Z., Liu Z., Xu W., Li X. (2024). (18)F-FDG PET/CT-based habitat radiomics combining stacking ensemble learning for predicting prognosis in hepatocellular carcinoma: A multi-center study. BMC Cancer.

[28] Tran D., Wang H., Torresani L., Ray J., LeCun Y., Paluri M. A Closer Look at Spatiotemporal Convolutions for Action Recognition. Proceedings of the 2018 IEEE/CVF Conference on Computer Vision and Pattern Recognition.

[29] Li J., Qiu Z., Zhang C., Chen S., Wang M., Meng Q., Lu H., Wei L., Lv H., Zhong W. (2023). ITHscore: Comprehensive quantification of intra-tumor heterogeneity in NSCLC by multi-scale radiomic features. Eur. Radiol..

[30] Huang Y., Wang X., Cao Y., Lan X., Hu X., Mou F., Chen H., Gong X., Li L., Tang S. (2025). Nomogram for Predicting Neoadjuvant Chemotherapy Response in Breast Cancer Using MRI-based Intratumoral Heterogeneity Quantification. Radiology.

[31] Li P., Wang X., Xu C., Liu C., Zheng C., Fulham M.J., Feng D., Wang L., Song S., Huang G. (2020). (18)F-FDG PET/CT radiomic predictors of pathologic complete response (pCR) to neoadjuvant chemotherapy in breast cancer patients. Eur. J. Nucl. Med. Mol. Imaging.

[32] Urso L., Manco L., Cittanti C., Adamantiadis S., Szilagyi K.E., Scribano G., Mindicini N., Carnevale A., Bartolomei M., Giganti M. (2025). 18F-FDG PET/CT radiomic analysis and artificial intelligence to predict pathological complete response after neoadjuvant chemotherapy in breast cancer patients. La. Radiol. Medica.

[33] Janssen L.M., Janse M.H.A., Penning de Vries B.B.L., van der Velden B.H.M., Wolters-van der Ben E.J.M., van den Bosch S.M., Sartori A., Jovelet C., Agterof M.J., Ten Bokkel Huinink D. (2024). Predicting response to neoadjuvant chemotherapy with liquid biopsies and multiparametric MRI in patients with breast cancer. NPJ Breast Cancer.

[34] Krishnamoorthy S., Morales E., Ashmanskas W.J., Werner M.E., Raj J., Matej S., Gao M., Vent T., Choi C.J., Maidment A.D.A. Imaging Performance of a dedicated Breast-PET-DBT scanner. Proceedings of the 2023 IEEE Nuclear Science Symposium, Medical Imaging Conference and International Symposium on Room-Temperature Semiconductor Detectors (NSS MIC RTSD).

[35] Mainta I.C., Sfakianaki I., Shiri I., Botsikas D., Garibotto V. (2023). The Clinical Added Value of Breast Cancer Imaging Using Hybrid PET/MR Imaging. Magn. Reson. Imaging Clin. N. Am..

[36] Fowler A.M., Miyake K.K., Nakamoto Y. (2024). Clinical Applications of Dedicated Breast Positron Emission Tomography. PET. Clin..

[37] Fanizzi A., Latorre A., Bavaro D.A., Bove S., Comes M.C., Di Benedetto E.F., Fadda F., La Forgia D., Giotta F., Palmiotti G. (2023). Prognostic power assessment of clinical parameters to predict neoadjuvant response therapy in HER2-positive breast cancer patients: A machine learning approach. Cancer Med..

[38] Gentile D., Martorana F., Karakatsanis A., Caruso F., Caruso M., Castiglione G., Di Grazia A., Pane F., Rizzo A., Vigneri P. (2024). Predictors of mastectomy in breast cancer patients with complete remission of primary tumor after neoadjuvant therapy: A retrospective study. Eur. J. Surg. Oncol..

[39] Xie T., Gong J., Zhao Q., Wu C., Wu S., Peng W., Gu Y. (2024). Development and validation of peritumoral vascular and intratumoral radiomics to predict pathologic complete responses to neoadjuvant chemotherapy in patients with triple-negative breast cancer. BMC Med. Imaging.

[40] Wang M., Chen W., Ren R., Lin Y., Tang J., Wu M. (2025). Comparative analysis of multi-zone peritumoral radiomics in breast cancer for predicting NAC response using ABVS-based deep learning models. Front. Oncol..

[41] Hou X., Chen K., Wan X., Luo H., Li X., Xu W. (2024). Intratumoral and peritumoral radiomics for preoperative prediction of neoadjuvant chemotherapy effect in breast cancer based on (18)F-FDG PET/CT. J. Cancer Res. Clin. Oncol..

[42] Hara K., Kataoka H., Satoh Y. Can Spatiotemporal 3D CNNs Retrace the History of 2D CNNs and ImageNet?. Proceedings of the 2018 IEEE/CVF Conference on Computer Vision and Pattern Recognition.

[43] Chen S., Ma K., Zheng Y. (2019). Med3D: Transfer Learning for 3D Medical Image Analysis. arXiv.

[44] Kim H.B., Tan H.Q., Nei W.L., Tan Y., Cai Y., Wang F. (2025). Impact of large language models and vision deep learning models in predicting neoadjuvant rectal score for rectal cancer treated with neoadjuvant chemoradiation. BMC Med. Imaging.

[45] Grapă C., Mocan T., Mocan L.P., Motofelea A., Stănciulescu R., Crăciun R., Vârciu A., Spârchez Z., Mocan T. (2025). Turning the Tide-Artificial Intelligence in the Evolving Landscape of Liver Cancer. Cancers.

